# Does breeding season variation affect evolution of a sexual signaling trait in a tropical lizard clade?

**DOI:** 10.1002/ece3.6167

**Published:** 2020-03-17

**Authors:** Levi N. Gray, Anthony J. Barley, David M. Hillis, Carlos J. Pavón‐Vázquez, Steven Poe, Brittney A. White

**Affiliations:** ^1^ Department of Biology University of New Mexico Albuquerque NM USA; ^2^ Department of Biology University of Hawai’i Honolulu HI USA; ^3^ Department of Integrative Biology University of Texas Austin TX USA; ^4^ Research School of Biology Australian National University Acton ACT Australia

**Keywords:** *Anolis*, dewlap, sensory drive, sexual selection, signaling trait evolution, species recognition

## Abstract

Sexually selected traits can be expected to increase in importance when the period of sexual behavior is constrained, such as in seasonally restricted breeders. *Anolis* lizard male dewlaps are classic examples of multifaceted signaling traits, with demonstrated intraspecific reproductive function reflected in courtship behavior. Fitch and Hillis found a correlation between dewlap size and seasonality in mainland *Anolis* using traditional statistical methods and suggested that seasonally restricted breeding seasons enhanced the differentiation of this signaling trait. Here, we present two tests of the Fitch–Hillis Hypothesis using new phylogenetic and morphological data sets for 44 species of Mexican *Anolis*. A significant relationship between dewlap size and seasonality is evident in phylogenetically uncorrected analyses but erodes once phylogeny is accounted for. This loss of strong statistical support for a relationship between a key aspect of dewlap morphology and seasonality also occurs within a species complex (*A. sericeus* group) that inhabits seasonal and aseasonal environments. Our results fail to support seasonality as a strong driver of evolution of *Anolis* dewlap size. We discuss the implications of our results and the difficulty of disentangling the strength of single mechanisms on trait evolution when multiple selection pressures are likely at play.

## Graphical abstract

Using new data sets, we retested support for a correlation between anole dewlap size and seasonality. Our results suggest that the previously recognized pattern of larger dewlaps in highly seasonal environments, independently verified in our study, is explained by shared evolutionary history.

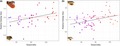

## 1 INTRODUCTION

Signaling traits such as nuptial color in fish and bird songs can greatly affect survival and fitness of individuals and thus can be used to understand how ecological and sexual selection drive phenotypic evolution (Cornwallis & Uller, 2010; Endler & Basolo, 1998). Postulated mechanisms for the evolution of these traits span numerous hypotheses of natural and sexual selection (Endler, 1992; Ryan & Rand, 1993). Since many speciose radiations exhibit signaling traits, these traits are also regularly identified as “key innovations” (Panhuis et al., 2001). Despite their apparent importance for evolution, determining selective drivers for signaling traits has proven difficult (Cornwallis & Uller, 2010).

Determining common mechanisms for the evolution of signaling traits is of great interest to biologists due in part to the vast interactions these traits initiate in nature. Signal trait evolution can be influenced not only by conspecifics via sexual selection (Darwin, 1871; Kirkpatrick, 1982; Ryan & Rand, 1993), but also by competitors (Grether, Losin, Anderson, & Okamoto, 2009; Rand & Williams, 1970) and predators (Endler, 1978; Tuttle & Ryan, 1982) through natural selection processes. This multitude of processes therefore has the potential for downstream ecological and evolutionary effects on organisms and ecosystems. For instance, hypotheses driving the variation in a trait might provide key insights to patterns of diversification via intraspecific processes (sensory drive; Endler, 1992) or community assembly via interspecific processes (species recognition; Rand & Williams, 1970). Thus, mechanisms driving the evolution of sexually selected traits are important components of understanding biological processes on multiple scales.

*Anolis* lizards are common research subjects in evolution (Losos, 2009). Among *Anolis* traits, dewlaps are perhaps the most discussed and least understood. Males of almost all ~400 anole species have dewlaps, which are flaps of gular skin used for species recognition, territorial behaviors, predator deterrence, and courtship (Losos, 2009). Dewlap variation—most notably in size, color, and display characteristics—is considerable in *Anolis*, and studies have characterized general patterns across species (Fitch & Hillis, 1984; Harrison & Poe, 2012; Ingram et al., 2016; Losos & Chu, 1998; Nicholson, Harmon, & Losos, 2007) and within‐species complexes (Driessens, Dehling, & Köhler, 2017; Ng, Landeen, Logsdon, & Glor, 2013; Vanhooydonck, Herrel, Meyers, & Irschick, 2009; White, Prado‐Irwin, & Gray, 2019). To date, attempts incorporating many species have largely failed to find strong support for any given hypothesis explaining the evolution of dewlap diversity (Losos & Chu, 1998; Nicholson et al., 2007).

Mechanisms suggested to play important roles in creating male dewlap variation include species recognition (Rand & Williams, 1970), sensory drive (Fleishman, 1992), and sexual selection (Fitch & Hillis, 1984). The species recognition hypothesis suggests that dewlap variation evolved as a means for recognition of conspecifics (Losos, 2009). This hypothesis garners support from the observation that many anole assemblages include multiple species with dewlaps that tend to vary greatly in size, pattern, or color (Losos, 2009; Rand & Williams, 1970). Quantitative support for species recognition driving evolution of dewlap traits, however, has been elusive. Losos and Chu (1998) and Nicholson et al. (2007) tested for dewlap correlation with environmental, lineage, and assemblage factors across *Anolis* and found weak (nonsignificant; Losos & Chu, 1998) support for sensory drive and no support for other hypotheses.

Sexual selection is the only hypothesis that has been supported for driving male dewlap size across a broad sample of anoles. Fitch and Hillis (1984) tested whether dewlap size correlates with length of the breeding season. Anole species living in seasonal areas were found to have shortened breeding seasons, while those in more aseasonal environments were found to breed throughout the year (Andrews & Rand, 1974; Fleming & Hooker, 1973; Fitch, 1972). The authors suggested that species with short breeding seasons experience intense sexual selection relative to species that occupy aseasonal environments and breed potentially continuously (Fitch & Hillis, 1984). Using data for 37 mainland species, they found that anoles in seasonal environments had larger dewlaps compared with species from aseasonal environments (Fitch & Hillis, 1984). Species with larger dewlaps also exhibited stronger male‐biased sexual size dimorphism, providing additional support for their hypothesis. *Anolis sericeus* (now considered a species complex; Gray et al., 2019; Lara‐Tufiño, Nieto‐Montes de Oca, Ramírez‐Bautista, & Gray, 2016), the one species found in both seasonal and aseasonal habitats, fit their interspecies pattern: A population they sampled from a seasonal environment possessed a larger dewlap than another from an aseasonal environment. Their findings of environmental seasonality affecting the evolution of a sexual signal could have important implications for sexual species that experience environmentally imposed restrictions on length of the breeding season. A similar effect of length of breeding season on a sexual trait has since been documented in harvestmen (Burns, Hedin, & Shultz, 2013), but to our knowledge has not been investigated in other systems.

An important advancement for testing hypotheses in evolutionary biology occurred with the development of methods for phylogenetic correction (Felsenstein, 1984; Harvey & Pagel, 1991). If male dewlap size variation violates assumptions of independence among anole species, evolutionary history should be taken into account before concluding support for the Fitch–Hillis Hypothesis. Based on their sampling, there is evidence phylogenetic history could be a confounding factor in analyses (Fitch & Hillis, 1984). For instance, most of the “seasonal” species (9 out of 16 species) sampled were from a single west Mexican clade that exhibits large dewlaps and occurs exclusively in seasonal areas (Poe et al., 2017). With a well‐sampled and strongly supported phylogeny (Poe et al., 2017), we can better address evolutionary questions via comparative methods that can account for phylogenetic nonindependence of traits like dewlap size.

Here, we aim to assess support for the Fitch–Hillis Hypothesis of temporal constraint driving evolution of a sexual signaling trait. Specifically, we test whether anole species experiencing short breeding periods have relatively larger male dewlaps than those that can breed for more extended temporal periods. We test this contention at two scales. First, we test for a relationship between seasonality and dewlap size across Mexican anole species using phylogenetic regression. Second, we test for this relationship within silky anoles (*Anolis sericeus* complex), the only species group that occurs throughout highly seasonal and aseasonal environments in the region.

## 2 METHODS

### 2.1 Data collection and measurements

We took digital photographs of male individuals collected between 2010 and 2018 throughout Mexico, spanning all habitat types inhabited by anoles and some representing noteworthy distribution records. Our samples were collected primarily in the winter (late November–February) and summer (June–September). LNG was involved in all dewlap photographs, either in taking the photograph or holding the animals. Dewlaps were extended by using forceps to pull the hyoid, and only dewlaps that were fully extended and aligned flat were included. Grid backgrounds were included in every dewlap photograph to scale measurements in all photographs. The dewlap for each photograph was outlined, and the area was calculated (mm^2^) on ImageJ (Schneider, Rasband, & Eliceiri, 2012). As a proxy for body size, we used head length in mm (HL; Ingram et al., 2016). Geographic coordinates were taken at collection sites.

**FIGURE 1:**
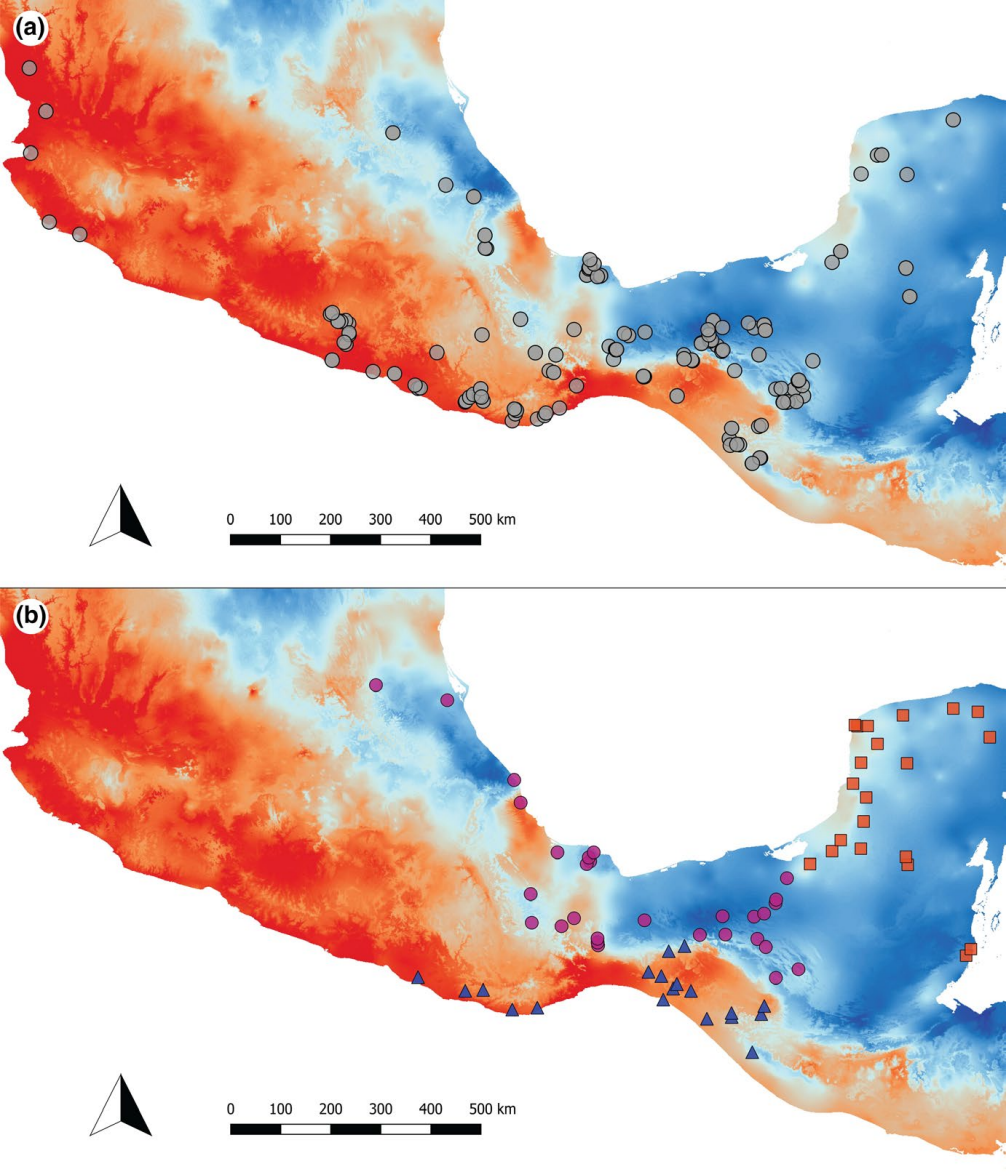
(a) Map of sampling localities. Background raster reflects seasonality of precipitation (BIO15), with warmer colors indicating high seasonality and cooler colors reflecting low seasonality. (b) Silky anole sampling colored by clade. The Pacific clade (blue) occurs in the Pacific versant of southern Mexico, the Caribbean clade (purple) occurs south and west of the Yucatan Peninsula in the Caribbean versant of Mexico, and the Yucatan clade (red) is only found in the Yucatan Peninsula. These three lineages form a clade excluding the rest of the silky anoles, which occur outside Mexico

In the original study, Fitch and Hillis (1984) used habitat type to categorize seasonal (desert, thorn scrub, deciduous forest, and dry coniferous forest) versus aseasonal (tropical rainforest and cloud forest) environments. Instead, we treated seasonality as a continuous variable. We extracted seasonality data from the seasonality of precipitation (BIO15) layer from WORLDCLIM (Hijmans, Cameron, Parra, Jones, & Jarvis, 2005) for each collection site using QGIS (QGIS Development Team, 2016). The BIO15 variable from WORLDCLIM is based on precipitation data and is used as a proxy for the length of the breeding season because *Anolis* lizards tend to have restricted breeding seasons tied to patterns of precipitation (Fitch & Hillis, 1984; Fleming & Hooker, 1973; Losos, 2009). The variable is calculated as the coefficient of variation in the monthly precipitation (O’Donnell & Ignizio, 2012). In effect, this variable reflects “evenness” of rainfall and, therefore, is directly linked to the length in a year in which conditions are acceptable for *Anolis* lizard reproduction (Fitch & Hillis, 1984; Fleming & Hooker, 1973). High BIO15 values, as reflected in the Pacific portion of the Isthmus of Tehuantepec (Figure 1), are related to strong decreases in precipitation during the dry season when reproduction is unable to occur in anoles (Fleming & Hooker, 1973). Exceptionally high values of BIO15 (above 100, commonly observed near the Pacific coast of southern and central Mexico) were investigated by O’Donnell and Ignizio (2012) and found to be regions where the variance of the precipitation “exceeded the average precipitation” and, therefore, represent good examples of environments where the breeding season is likely to be truncated and lead to the heightened levels of sexual selection discussed by Fitch and Hillis (1984). Lower values of BIO15 are seen in areas that tend to be consistently wet such as in lowland Caribbean rainforests and cloud forests (Figure 1) and therefore offer a longer period for reproduction, as described by Fitch and Hillis (1984; see also Fitch, 1972).

### 2.2 Species comparisons

Mexican anole taxonomy has a long history of uncertainty (Lieb, 2001; Nieto‐Montes de Oca, Poe, Scarpetta, Gray, & Lieb, 2013). We analyzed all Mexican species for which we had data and for which previous research supports their validity (*n* = 44; Appendix 1; Tables A1 and A2). To account for phylogeny, we used a recently published phylogeny (the maximum clade credibility tree for anoles from Poe et al., 2017), trimming the tips to match our sampled species using the “ape” package in R (Paradis et al., 2004).

For each species, we averaged male dewlap size and seasonality values from sampled localities. To verify consistency in both dewlap size and seasonality values within species, we calculated the intraclass correlation coefficient (ICC) for species for which we had at least 5 samples using the “irr” package in R. We calculated ICCs by randomly selecting 5 samples for each species before running analyses. Because body size is a confounding variable for dewlap size (Losos & Chu, 1998), we ran a phylogenetic regression using the “ape” and “nmle” packages (Pinheiro et al., 2019) in R (R Core Team, 2014) on log‐transformed dewlap size with log‐transformed HL for all species (Schoener, 1970). We used the residual values from that regression as relative dewlap size for each species. We then performed phylogenetic regression on relative dewlap size against seasonality. To assess the strength of phylogenetic signal of BIO15, we calculated Pagel's λ and Blomberg's K using the “ape” and “phytools” packages in R version 3.6.0, respectively (Revell, 2012). We also performed these analyses using the “stats” package for standard (i.e., phylogenetically uncorrected) Ordinary Least Squares regressions.

In Mexico, the *Anolis sericeus* group (silky anoles; considered by Fitch & Hillis[, 1984] to be a single species) consists of three divergent clades (referred to here as Pacific, Caribbean, and Yucatan) that may be separate species (Gray et al., 2019; Figure 1, Appendix 1). A phylogenetic regression was not possible for these analyses, as molecular data are absent for a number of the sampled populations (Gray et al., 2019). We averaged male dewlap size for specimens from each locality and performed a standard OLS regression of dewlap size and head length using all silky anole localities. Using residuals from that regression as measures for relative dewlap size, we then regressed those values against seasonality. Subsequently, we performed another regression analysis using localities from only the Pacific and Caribbean clades. The Yucatan population is diagnosed by small male dewlaps (Lara‐Tufiño et al., 2016). By removing these forms, we tested whether the Yucatan is strongly influencing the results of the analysis when the group is analyzed as a whole. We also ran each lineage on its own to test for signal within each group.

We performed a series of alternative analyses to explore consistency of our results and test support for the Fitch–Hillis Hypothesis. These included using HL as a covariate in the regression, using absolute values of dewlap size (largest sample for each species and average dewlap size), and testing season of collection as a covariate. Details for these analyses are in Appendix 1.

## 3 RESULTS

We compiled data for 230 adult male dewlaps representing 41 species for our interspecies analyses. Sampling within species ranged from 1 to 38 individuals from 1 to 22 localities. Within‐species variation in dewlap size and seasonality was minimal (Table A1), reflected in ICC scores of 0.768 in dewlap size (*n* = 14 species; *p* < .001) and 0.824 in seasonality (*n* = 7 species; *p* < .001). Data files, including those used in alternate analyses, are available on Dryad.

**FIGURE 2:**
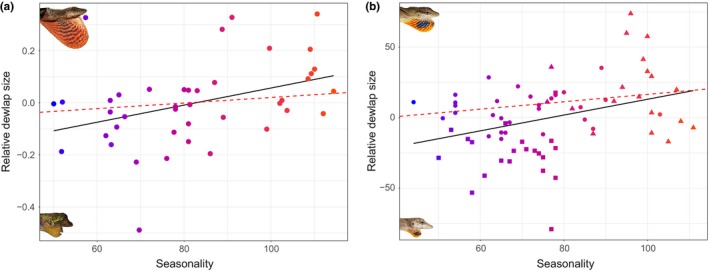
(a) Plot showing standard ordinary least squares (OLS) regression (black line; *p* = .01537) and phylogenetic least squares regression (dotted red line; *p* = .5519) of broad analyses. (b) Plot showing OLS regression of all silky anole populations (black line; *p* = .002742) and only Pacific and Caribbean silky anole populations (dotted red line; *p* = .1059). Plotted points are colored by seasonality, with warmer colors denoting stronger seasonality environments

Our standard OLS regression resulted in a significant positive relationship between relative dewlap size and seasonality (*p* = .01537; *F*‐statistic = 6.426, DF = 39; Figure 2), consistent with results from Fitch and Hillis (1984). However, accounting for phylogenetic relationships rendered the relationship nonsignificant (*p* = .5519; Figure 2). Pagel's λ was 1.089, and Blomberg's K for BIO15 was 1.039, indicating strong phylogenetic signal in seasonality. These results were in agreement with all alternate PGLS analyses in failing to find a significant relationship between dewlap size and seasonality (see Appendix 1 for more details on analyses and results).

Sampling for the silky anoles included 153 adult males representing 69 populations/localities (19 Pacific, 29 Caribbean, and 21 Yucatan). OLS regression on the entire group produced a significant positive relationship between relative dewlap size and seasonality (*p* = .002742, adjusted *r* ^2^ = 0.1131, *F*‐statistic = 9.676, DF = 67; Figure 2). The regression incorporating only the Pacific and Caribbean lineages, however, is nonsignificant for relative dewlap size and seasonality (*p* = .1059; Figure 2). Each of the single lineage analyses resulted in nonsignificant results (Appendix 1; Figure A1).

## 4 DISCUSSION

In each of our analyses, the effect of seasonality on male dewlap size was small enough that we could not confidently distinguish it from no effect once phylogeny was taken into account. Our results regarding phylogenetic signal for seasonality suggest that phylogenetic inertia is a reasonable alternative hypothesis for the pattern observed by Fitch and Hillis (1984). Results for the silky anole clade strongly depended on the exclusion or inclusion of the Yucatan lineage, whose individuals possess a small dewlap and inhabit a primarily aseasonal region. The Pacific and Caribbean lineages span the most and least seasonal environments inhabited by the group and do not show a strong correlation between seasonality and dewlap size, nor do analyses of single lineages (Figure A1). Individuals in the Caribbean lineage (sister to the Yucatan; Gray et al., 2019) do not have a reduced dewlap and occur in statistically indistinguishable seasonality environments (Table 1). If seasonality affects the evolution of dewlap size in silky anoles, we would expect to see reduced dewlaps in both lineages found in relatively aseasonal environments or a significant increase in dewlap size in the lineage inhabiting seasonal environments, neither of which occurs.

**TABLE 1:** Silky anole sampling with average and range of seasonality experienced by each lineage. The Caribbean and Yucatan lineages occur in environments with indistinguishable seasonality (*t* test, *p* = .5896)

Lineage	Sample number	Number of localities	Average seasonality	Range of seasonality
Caribbean	63	29	69.9	44–103
Pacific	44	19	96.3	76–111
Yucatan	46	21	68	50–78

Our results and those of others (Losos & Chu, 1998; Nicholson et al., 2007) suggest that sexual selection, species recognition, and sensory drive hypotheses are not strongly supported as sole explanations driving dewlap size in large multispecies analyses of anoles. However, dewlaps vary along multiple axes, including color and display mechanics, which may jointly shape phenotypic evolution. Variability in dewlap color, pattern, size, and use is considerable in *Anolis*, and unlikely to be the result of a single selective force (Losos & Chu, 1998). Experimental studies may disentangle causal mechanisms for dewlap evolution (Driessens et al., 2017; Leal & Fleishman, 2003), though in‐depth studies of the widespread species *A. sagrei* have had limited success in this endeavor (Baeckens, Driessens, & Damme, 2018). As additional data on other aspects of dewlap traits (color, UV reflectance, pattern, display characteristics, etc.), phylogenetic relationships, and natural history become available, testing more complex hypotheses may be feasible.

We note that while none of our analyses that take evolutionary history into account found a significant relationship, our results do exhibit positive slopes (Figure 2). These results could indicate a minor effect of seasonality on dewlap size in the anoles in our study that is too weak for our current methods and sampling to disentangle (we also found strong phylogenetic signal for BIO15, our proxy for seasonality). It is also possible there have not been enough shifts between seasonal and aseasonal environments in Mexican anoles to detect an effect. And though we found consistency in dewlap sizes within species, one species outside the tropics is known to exhibit plasticity in dewlap size (*Anolis carolinensis*; Lailvaux, Leifer, Kircher, & Johnson, 2015). This type of plasticity, if present in tropical anoles, might obscure a relationship between dewlap size and seasonality, particularly when within‐species sampling is low. However, plasticity in *A. carolinensis* is associated with skin elasticity and a lack of use during the winter when the lizards are inactive. Anoles in tropical regions are still active outside of the breeding season (Fleming & Hooker, 1973; Henderson & Fitch, 1975; Gray & White pers. obs.). We also found no effect of season of collection affecting dewlap size in our data set when we tested for this explicitly (Appendix 1), though the possibility of plasticity in tropical anoles awaits more thorough investigation.

Looking closely at patterns within taxonomic groups casts more doubt on the strength of any putative effect of the length of breeding season on dewlap size in Mexican anoles. Among species in the monophyletic west Mexican anole clade, which we sampled entirely in this study (Poe et al., 2017), dewlap sizes tend to be large and species tend to occur in seasonal environments. However, the species with the largest dewlap in the group, *Anolis macrinii*, also occurs in the least seasonal environment (Table A1, Appendix 1). Another large‐dewlapped species in our study, the semiaquatic *A. barkeri*, occurs in some of the least seasonal environments of all Mexican anoles. Since other semiaquatic species are also known to have large dewlaps in Central America (Ingram et al., 2016), it may be tempting to assume that the semiaquatic lifestyle leads to large dewlaps. However, the Cuban species *A. vermiculatus* may be the most aquatic anole and is one of the only anole species that entirely lacks a dewlap. These observations raise two important points. First, they demonstrate the need for large data sets to avoid drawing conclusions based on small sample sizes. Second, that a number of factors likely contribute to dewlap trait variation in anoles as expected for complex signaling traits (Endler, 1992).

If length of the breeding season does not explain evolution of dewlap size in anoles, will any other single variable explain dewlap size variation across a broad sampling of anole species? We have our doubts, as mechanisms shaping the evolution of sexual traits are often difficult to determine in empirical systems (Cornwallis & Uller, 2010). Additionally, the complexity of sexual trait evolution is such that testing a single mechanism is likely to fail (Endler, 1992). We suspect demographic and natural history characteristics may explain some of the dewlap size variation observed among anole species. For instance, sensory drive is likely an important factor in some anole species (Leal & Fleishman, 2003) but its strength as a driver for dewlap evolution is partly dependent on the likelihood that a particular female will encounter multiple males or will have a preference for males exhibiting larger or more visible dewlaps. One could also envision a scenario in which the opposite of the Fitch–Hillis Hypothesis is true: Species/populations with shorter breeding seasons could yield weaker selection on male dewlap size due to limited time for females to seek out preferred males. Unfortunately, data on abundance and relevant natural history traits are lacking for the vast majority of anole species. Taxonomic groups without some of these complications or with more complete natural history data may be better suited for testing the effect of temporal constraint on signaling trait evolution. Groups containing variation in length of breeding season and in signaling traits are common and can provide further resolution as to the efficacy of the hypothesis moving forward. Advances in understanding female choice since the Fitch and Hillis study (1984) have only strengthened the potential relevance of their hypothesis; females commonly “prefer traits of greater quantity” (Andersson, 1994; Ryan & Keddy‐Hector, 1992). Examples of putative study subjects span a broad swath of animal life, including spiders (*Habronattus pugillis*, Maddison & McMahon, 2000), insects (odonates, Serrano‐Meneses, Cordoba‐Aguilar, Azpilicueta‐Amorin, González‐Soriano, & Szekely, 2008), frogs (*Acris crepitans*, Ryan & Wilczynski, 1991), birds (*Ficedula hypoleuca*, Järvi, Røsskaft, Bakken, & Zumsteg, 1987), and fish (Malawi cichlids, Marsh, Marsh, & Ribbink, 1986). The availability of natural history data is without question the biggest limitation in testing the Fitch–Hillis Hypothesis and other hypotheses addressing evolutionary processes in empirical systems.

Given our results in these analyses, currently there is limited support for temporal constraint driving evolution of sexual traits (but see Burns et al., 2013). Although Fitch and Hillis (1984) suggested dewlap color could be a mitigating factor for size in anoles, our preliminary data do not support this idea. Species with small dewlaps in seasonal environments do not have categorically “brighter” dewlaps than those with large dewlaps (Appendix 1; Table A1), and incorporating color into future analyses will be challenging. Perhaps approaches focusing on additional factors (UV reflectance, display characteristics, etc.) and overall signal conspicuousness (Endler, 1992) or additional groups of anoles distributed across seasonal and aseasonal environments will find stronger support for a modification of the Fitch–Hillis Hypothesis in anoles. We hope that our study will encourage further research in testing the effect of temporal constraint on sexually selected traits in other groups, as the Fitch–Hillis Hypothesis remains theoretically plausible and untested in a number of appropriate sexual signaling systems.

## CONFLICTS OF INTEREST

The authors declare no conflicts of interest.

## AUTHOR CONTRIBUTIONS

LNG conceived the study. LNG, AJB, CJPV, BAW, and SP collected samples and took photographs. LNG collected data, and LNG, AJB, and BAW performed analyses. All authors wrote the manuscript and discussed result interpretation. All authors are accountable for the content and approved the final version of this manuscript.

## Supporting information

Supplementary Material

## Data Availability

Data have been deposited at the Dryad digital repository: https://doi.org/10.5061/dryad.80gb5mknk.

## Appendix 1

### Taxonomic decisions

#### Excluded taxa in broad species comparison

We decided it would be premature to recognize several recently‐described Mexican anole species that lacked strong evidence supporting their recognition. Including taxa that do not properly reflect species‐level diversity could potentially bias the results of our analyses. The populations we are not recognizing as separate species are indistinguishable by dewlap size and color and occur in the same seasonality environments as the species from which they were suggested to be split from.

We excluded taxa that were described primarily on the basis of differences in hemipenial morphology between populations (Köhler et al., 2010, 2014; Köhler & Veseley, 2010). The primary justification for describing these taxa (despite continuous distribution of populations) was that hemipenial traits should be associated with reproductive isolation. In the species groups that have been investigated with multiple lines of molecular and/or morphological evidence, this hypothesis has not been supported. For instance, there are now several documented cases of within‐species and within‐population variation in hemipenial morphology (Gray et al., 2019; Köhler et al., 2012; Lara‐Tufiño et al., 2016; Phillips et al., 2015). Finding variation in hemipenial morphology within species is to be expected due to high rates of hemipenial evolution (Klaczko et al., 2015) and the resulting likelihood of multiple hemipenial forms to exist prior to speciation (as expected with other traits not associated with reproductive isolation; Lara‐Tufiño et al., 2016). Gray et al. (2019) used a large genomic (restriction site‐associated DNA; RAD) data set to infer phylogeographic relationships in the silky anoles (*Anolis sericeus* complex) and found no association between population divergence and hemipenial morphology. The taxa described based primarily on hemipenial morphology that we excluded from analyses, *A. carlliebi* and *A. sacamecatensis* (Köhler et al., 2014), were poorly sampled throughout the group's continuous distribution, making it difficult to make a strong case in support of recognizing the putative species as valid at this point in time.

We also excluded taxa that were described solely on the basis of varying levels of divergence among mitochondrial haplotypes (Köhler et al., 2014). There is an abundance of evidence that mtDNA haplotypes can exhibit strong patterns of divergence without barriers to gene flow (Funk & Omland, 2003; Irwin, 2002; Petit & Excoffier, 2009), and anole systems have been known to show significant mitochondrial divergence between freely‐interbreeding populations (Ng & Glor, 2011; Ng et al., 2016; Thorpe et al., 2008, 2010). Anole populations delimited as separate species by Köhler et al. (2014) exhibit far lower levels of mtDNA divergence than other populations known to maintain evolutionary cohesion (Ng & Glor, 2011; Thorpe et. al., 2008). Köhler et al. (2014) also lacked sampling from near putative contact zones of the populations they described as separate species, which causes further difficulty in interpreting their results. A standard isolation‐by‐distance model could potentially explain the resulting phylogenetic structure in their analyses, and in the absence of evidence for reduced or absent gene flow it would be questionable to assume the continuously distributed populations are on independent trajectories.

For a summary on evidence for and against species not recognized in our study, see Table A2.

#### Silky anole sampling

We grouped populations by clades recovered via phylogenetic analysis of a large multilocus genomic data set (Gray et al., 2019) rather than species delimited using hemipenial morphology (Köhler & Vesely, 2010). The molecular data used in that study consists of over 500 restriction‐site associated DNA (RAD) markers and attained strong resolution for clades within the silky anole group. The populations present in Mexico represent a monophyletic group that is deeply divergent from populations in Guatemala, Honduras, and to the southeast. Only the Yucatan lineage has been shown to be morphologically distinct. The name *Anolis ustus* has been resurrected for this lineage, which can be diagnosed by a smaller dewlap in males (relative to other silky anoles) and a slightly larger dewlap in females (Lara‐Tufiño et al., 2016).

### Alternate analyses

We also tested for correlation between dewlap size and seasonality with head length (HL) as a covariate. The results for these additional analyses were similar to our analyses testing relative dewlap size versus seasonality (*p* = .0147, adjusted R‐squared = 0.6821, *F*‐statistic = 43.91, 38 degrees of freedom).

Results of the phylogenetically corrected interspecies analysis using HL as a covariate were also similar to the analyses presented in the main text (*p* = .5567). Pagel's Lambda model using log HL as a covariate resulted in a value of 1.089 (the same Lambda value from the analyses in the main body of the manuscript).

For the silky anole analyses, using HL as a covariate on the whole group resulted in significant statistical support (*p* = .00657, adjusted r‐squared = 0.5321, *F*‐statistic = 37.39, 62 degrees of freedom). Removing the Yucatan lineage again resulted in a non‐significant result: *p* = .152 (*F*‐statistic = 36.72; 44 degrees of freedom). None of the analyses of individual lineages yielded significant results (Caribbean, *p* = .623; Pacific, *p* = .5836; Yucatan, *p* = .653).

Rather than relative dewlap size, it is possible that absolute dewlap size is more important for signal efficacy. Analyses using maximum absolute dewlap size (largest among our samples) on the interspecies data set yielded nonsignificant relationships between dewlap size and seasonality (OLS: *p* = .9129, adjusted r‐squared=−0.02532, *F*‐statistic = 0.01213, 39 degrees of freedom; PGLS: *p* = .3676). Using average dewlap size rather than maximum attained the same result (OLS: *p* = .6569, adjusted *r*‐squared = −0.0204, *F*‐statistic = 0.2003, 39 degrees of freedom; PGLS: *p* = .9494).

Lailvaux et al.’s (2015) study demonstrating seasonal plasticity in dewlap size in *Anolis carolinensis*, if widespread in the genus, could be a confounding factor in our analyses. Our ICC analyses indicate consistency in dewlap sizes within species (including individuals collected across multiple seasons, which suggests seasonal plasticity is not widespread in species in our data set).

In another attempt to verify whether seasonal plasticity could be driving the relationships, we ran analyses on the interspecies data set using season of collection as a covariate. We categorized season of collection as either Wet or Dry for each sample and pared down the data set so that only samples from the best‐sampled season were used. Samples categorized as “Dry” season were from late November to February, which is within the period of low rainfall (Janzen, 1967; Müller, 1996) in tropical Mexico. We then ran OLS and PGLS on this data set using season of collection as covariates. The OLS results were marginally significant (*p* = .04904, adjusted r‐squared = 0.1018, *F*‐statistic = 3.267), while the results of the PGLS analysis were nonsignificant (*p* = .6049). Season was not found to be a significant factor in either OLS (*p* = .3149) or PGLS analyses (*p* = .2615).
