# Supplementary material for: Does breeding season variation affect evolution of a sexual signaling trait in a tropical lizard clade?
Source: Ecol Evol. 2020 Mar 17;10(8):3738–46. doi: 10.1002/ece3.6167 (PMC7160170; doi:10.1002/ece3.6167)

**Table A1**

Table providing details on per species sampling, range in dewlap size, seasonality of sampled localities, and dewlap color. For dewlap color, up to three colors are listed: primary color, secondary color, and tertiary color are ranked by proportion of each color in the dewlap.

| *Anolis* Species | No. of Samples | No. of Localities | Range logdsize | Range Seasonality | Color |
| --- | --- | --- | --- | --- | --- |
| *alvarezdeltoroi* | 3 | 2 | 2.51-2.62 | 55-71 | Red |
| *barkeri* | 7 | 4 | 2.69-3.13 | 43-66 | Red/purple/white |
| *beckeri* | 8 | 5 | 1.68-2.36 | 44-54 | Pink |
| *biporcatus* | 2 | 2 | 2.62-2.76 | 64-74 | Blue/orange/white |
| *boulengerianus* | 11 | 9 | 1.94-2.5 | 92-115 | Orange/yellow |
| *campbelli* | 2 | 1 | 2.4 | 65 | Pink |
| *capito* | 3 | 3 | 2.01-2.22 | 68-71 | Yellow |
| *compressicauda* | 3 | 3 | 2.37-2.41 | 44-57 | Purple/yellow |
| *crassulus* | 4 | 3 | 2.13-2.47 | 75-85 | Orange |
| *cristifer* | 1 | 1 | 2.27 | 86 | Red |
| *cuprinus* | 4 | 1 | 2.17-2.41 | 87 | Purple |
| *cymbops* | 1 | 1 | 2.37 | 79 | Pink |
| *dollfusianus* | 3 | 2 | 2.05-2.07 | 75-81 | Yellow |
| *duellmani* | 7 | 3 | 1.97-2.37 | 63 | Purple |
| *dunni* | 2 | 2 | 2.29-2.48 | 106-111 | Red/yellow |
| *gadovii* | 1 | 1 | 2.78 | 108 | Purple/pink |
| *hobartsmithi* | 6 | 4 | 2.2-2.37 | 48-52 | Purple |
| *laeviventris* | 16 | 12 | 1.71-2.17 | 50-99 | White |
| *lemurinus* | 38 | 15 | 1.84-2.52 | 53-76 | Red/orange |
| *liogaster* | 2 | 2 | 2.21-2.52 | 102-103 | Purple |
| *macrinii* | 1 | 1 | 3.14 | 90 | Orange/white |
| *matudai* | 3 | 3 | 2.24-2.31 | 83-99 | Purple/yellow |
| *megapholidotus* | 2 | 2 | 1.94-2 | 110-111 | Pink |
| *microlepidotus* | 1 | 1 | 2.28 | 102 | Orange/yellow |
| *milleri* | 1 | 1 | 2 | 81 | Pink-purple |
| *naufragus* | 6 | 3 | 2.15-2.38 | 68-78 | Orange-red |
| *nebuloides* | 15 | 11 | 1.97-2.63 | 93-109 | Pink |
| *nebulosus* | 8 | 8 | 1.72-2.28 | 110-126 | Orange |
| *omiltemanus* | 4 | 3 | 2.04-2.29 | 102-104 | Orange |
| *parvicirculatus* | 2 | 2 | 2.38-2.41 | 80-83 | Red/orange |
| *petersi* | 4 | 2 | 2.14-2.66 | 63-88 | Red/black |
| *peucephilus* | 1 | 1 | 1.93 | 99 | Orange |
| *quercorum* | 6 | 4 | 2.16-2.47 | 87-90 | Pink |
| *rodriguezii* | 33 | 22 | 1.59-2.03 | 43-89 | Orange-yellow/red |
| *rubiginosus* | 1 | 1 | 2.04 | 81 | Pink |
| *schiedii* | 2 | 1 | 2.4-2.44 | 78 | Orange |
| *serranoi* | 3 | 3 | 2.51-2.61 | 75-88 | Red/black |
| *subocularis* | 6 | 6 | 2.46-2.87 | 107-113 | Pink/yellow |
| *taylori* | 2 | 1 | 2.64-2.69 | 111 | Red/mint |
| *tropidonotus* | 3 | 2 | 2.09-2.3 | 65 | Yellow/red |
| *uniformis* | 5 | 4 | 1.9-2.12 | 53-66 | Pink/purple |

**Table A2**

Table summarizing evidence for and against recently described species not recognized in this study.

| Species not recognized | Synonymous with | Evidence for validity | Evidence against validity |
| --- | --- | --- | --- |
| *Anolis nietoi* | *Anolis nebuloides* | mtDNA clustering/hemipenial morphology | Phylogenetic trees not consistent among markers; not diagnosable morphologically and continuous distribution of population does not suggest reproductive isolation |
| *Anolis stevepoei* | *Anolis nebuloides* | mtDNA clustering/hemipenial morphology | Phylogenetic trees not consistent among markers; not diagnosable morphologically and continuous distribution of population does not suggest reproductive isolation |
| *Anolis zapotecorum* | *Anolis nebuloides* | mtDNA clustering/hemipenial morphology | Phylogenetic trees not consistent among markers; not diagnosable morphologically and continuous distribution of population does not suggest reproductive isolation |
| *Anolis carlliebi* | *Anolis quercorum* | mtDNA clustering/hemipenial morphology | Not diagnosable externally and only slight hemipenial differences. Molecular sampling missing important/intermediate localities. |
| *Anolis sacamecatensis* | *Anolis quercorum* | mtDNA clustering/hemipenial morphology | Not diagnosable externally and only slight hemipenial differences. Molecular sampling missing important/intermediate localities. |
| *Anolis immaculogularis* | *Anolis subocularis* | Distinct in dewlap coloration from one species and in mtDNA divergence from another species | Phylogenetic trees show a clustering of a mix of *immaculogularis* and *subocularis*; the population described is not distinct in morphology or in mtDNA. |

**Figure A1**

Figure showing best-fit Ordinary Least Square regression lines for each silky anole lineage. Red squares represent the Yucatan lineage, purple circles represent the Caribbean lineage, and blue triangles represent the Pacific lineage. The Caribbean regression line shows a positive slope, unlike the other two groups, but with a p-value of 0.623 it remains nonsignificant.


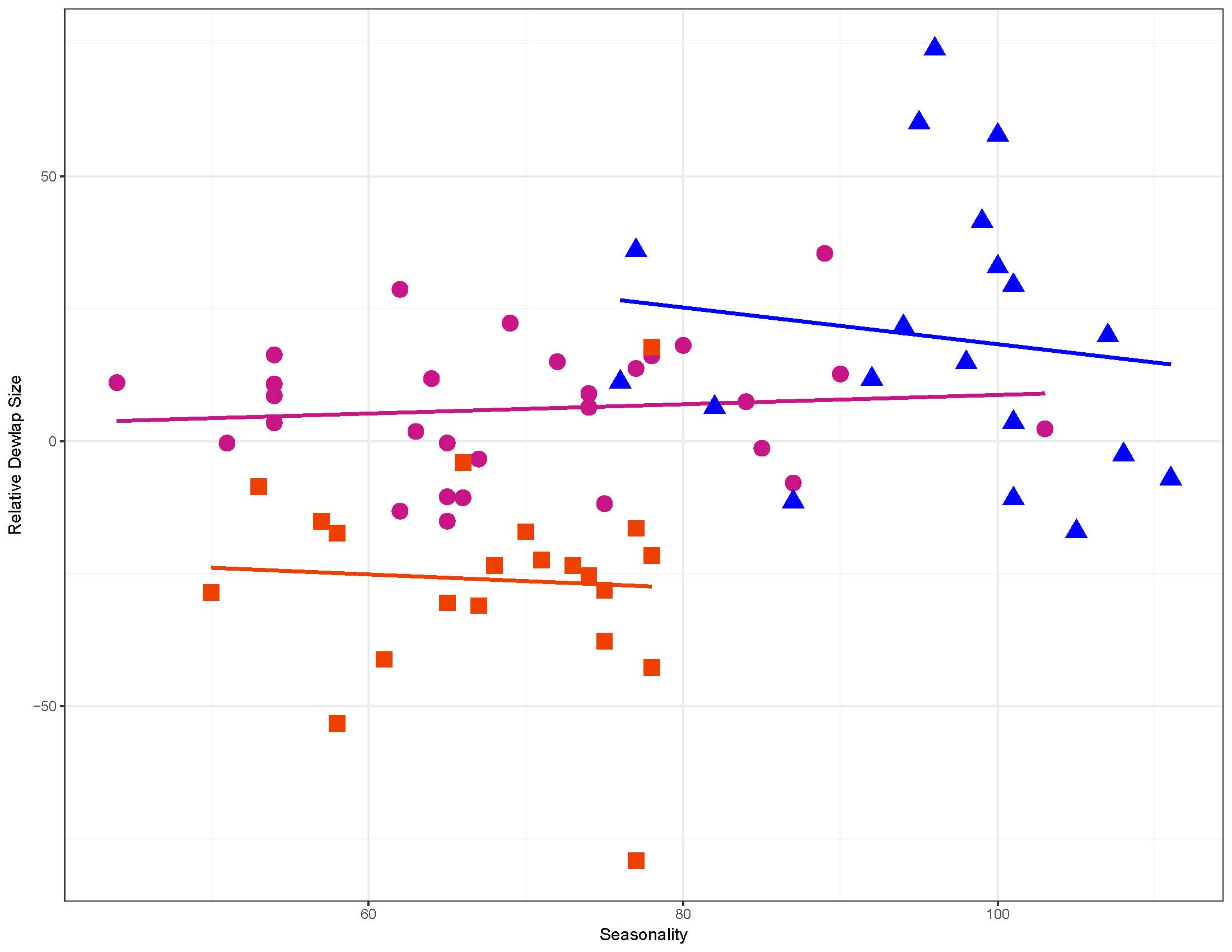

Supplement: Supplementary file 1 — Supplementary Material [file ECE3-10-3738-s001.docx]
